# Association of neuropathy, sarcopenia and physical function in acute lymphoblastic leukemia survivors with chemotherapy alone

**DOI:** 10.1007/s11764-026-02007-3

**Published:** 2026-03-26

**Authors:** Martin Kaj Fridh, Mengqi Xing, Sedigheh Mirzaei, David Mizrahi, Hanne Bækgaard Larsen, Stephanie B. Dixon, Nicholas S. Phillips, Kevin R. Krull, Hiroto Inaba, Ching-Hon Pui, Seth E. Karol, Angela Delaney, Sue C. Kaste, Melissa M. Hudson, Kirsten K. Ness

**Affiliations:** 1 The Daffodil Centre, The University of Sydney, Sydney, Australia; 2 Faculty of Health Sciences, University of Copenhagen, Copenhagen, Denmark; 3 Department of Biostatistics, St. Jude Children’s Research Hospital, Memphis, TN USA; 4 Department of Epidemiology and Cancer Control, St. Jude Children’s Research Hospital, 262 Danny Thomas Place, MS-735, Memphis, 38105 TN USA; 5 Department of Oncology, St. Jude Children’s Research Hospital, Memphis, TN USA; 6 Department of Psychology and Biobehavioral Sciences, St. Jude Children’s Research Hospital, Memphis, TN USA; 7 Department of Endocrinology, St. Jude Children’s Research Hospital, Memphis, TN USA; 8 Department of Radiology, St. Jude Children’s Research Hospital, Memphis, TN USA; 9 Department of Pediatrics and Adolescent Medicine, The Juliane Marie Center, Copenhagen University Hospital-Rigshospitalet, Copenhagen, Denmark

**Keywords:** Acute lymphoblastic leukemia, Survivors, Neuropathy, Sarcopenia, Physical function

## Abstract

**Background:**

Despite excellent survival and elimination of cranial radiation, patients treated for acute lymphoblastic leukemia (ALL) in childhood remain at increased risk for chronic conditions, including peripheral neuropathy and sarcopenia. This study aimed to evaluate the association between peripheral neuropathy and sarcopenia in survivors of childhood ALL without prior cranial radiation exposure. Additionally, we explore the effects of neuropathy and sarcopenia on physical function and exercise behavior.

**Methods:**

We included survivors of childhood ALL diagnosed between 1962 and 2012, aged ≥18 years without a history of cranial radiation from the St. Jude Lifetime Cohort Study (SJLIFE). Peripheral neuropathy was assessed using the Modified Total Neuropathy Score (mTNS). Sarcopenia was defined by low muscle mass (dual X-ray absorptiometry) and muscle weakness, with muscle strength assessed using hand grip and quadriceps strength tests. Physical function was evaluated with the Timed-Up-and-Go test and a 50-foot walk test. Physical activity was self-reported via the NHANES Physical Activity Questionnaire. Statistical analyses, including modified Poisson regression, were performed to examine associations.

**Results:**

Among 537 survivors (median age: 28 years, range 18–52), 31.7% had peripheral neuropathy, 19.0% had reduced muscle strength, and 21.6% had reduced relative lean muscle mass. Neuropathy was significantly associated with impaired gait speed (RR: 1.31, 95% CI 1.01 to 1.7). Sarcopenia, particularly impaired muscle strength, was associated with impaired mobility (RR: 2.99, 95% CI 1.91 to 4.68), gait speed (RR: 1.45, 95% CI 1.06 to 1.97), and <150 min/week of moderate or vigorous physical activity (RR 1.43, 95% CI 1.04 to 1.95).

**Conclusion:**

Peripheral neuropathy and sarcopenia significantly impact physical function in childhood ALL survivors, with impaired muscle strength emerging as a key determinant of mobility limitations. These findings emphasize the importance of targeted interventions, particularly strengthening exercise, to improve functional outcomes in this vulnerable population. Future research should focus on developing evidence-based rehabilitation strategies to enhance long-term survivorship care.

**Clinical impact:**

Evaluation of muscle strength, lean mass, and gait performance may facilitate early identification of survivors at risk for functional decline and premature aging.

**Supplementary Information:**

The online version contains supplementary material available at https://doi.org/10.1007/s11764-026-02007-3.

## Introduction

Advances in the medical treatment of childhood acute lymphoblastic leukemia (ALL), including the elimination of cranial radiation for central nervous system prophylaxis in nearly all children, have resulted in 5-year survival rates above 90% [1] and reductions in late mortality [2, 3]. Nevertheless, ALL treatment is not without consequences [4, 5]. Childhood ALL survivors treated more recently are at particular risk for musculoskeletal and neurosensory impairments [4–8]. Musculoskeletal impairments include reduced muscle strength [9–13] and low skeletal muscle mass [14–21], placing survivors at risk of developing sarcopenia, a musculoskeletal disorder characterized by loss of muscle mass and strength and typically associated with aging, early in their lives. In the general population, sarcopenia is associated with an increased risk of adverse outcomes such as falls and fractures, difficulty performing daily activities, and loss of independence [22–24]. It is further associated with low bone mineral density, cardiometabolic disease, and cognitive impairments, affecting quality of life and mortality [23, 25–28].

Chemotherapy-induced peripheral neuropathy (CIPN) is also a potentially long-lasting adverse effect of ALL treatment [29]. While neuropathy symptoms are rarely life-threatening, they are often severe enough to impact physical function, limiting mobility, and participation in daily activities, which can eventually impact the quality of life [30–33]. ALL survivors have documented peripheral electrophysiological impairments, including reduced amplitude of sural sensory nerve action potentials and reduced amplitude and prolonged latency of compound muscle action potentials [31, 34–37]. These data suggest suboptimal peripheral nerve function, reduced number of functioning axons, and loss of motor units. Abnormal feedback during movement and loss of motor units likely contribute to the skeletal muscle atrophy and weakness observed during and after ALL therapy [12, 13]. However, the contributions of persistent CIPN in the development of sarcopenia and associated muscle weakness in ALL survivors remains poorly understood. Therefore, we aimed to evaluate the association between peripheral neuropathy and sarcopenia in childhood ALL survivors without previous cranial radiation exposure. Additionally, we aimed to evaluate the effects of peripheral neuropathy and sarcopenia on physical function (mobility and gait speed) and exercise behavior (physical activity) in adult survivors of childhood ALL.

## Materials and methods

### Study participants

Participants are members of the St. Jude Lifetime Cohort (SJLIFE) [38], a study that aims to facilitate the longitudinal evaluation of health outcomes among survivors of childhood cancer treated at St. Jude Children’s Research Hospital (SJCRH). Potentially eligible cancer survivors previously treated for childhood cancer are invited to return to campus, where they complete clinical evaluation, a core battery of laboratory testing, imaging, performance testing, and questionnaires. For this study, eligible participants were those diagnosed with ALL between 1962 and 2012, who survived ≥5 years from diagnosis, were ≥18 years old at assessment, and had no history of cranial radiation exposure. We excluded survivors diagnosed with hemiplegia, paralysis, or a congenital or acquired (e.g., amputation, closed head injury) condition associated with sensory or motor function and participants with missing outcome data. The Institutional Review Board approved the protocol and study documents. Participants provided written informed consent before completing study measures. All participants received per-day financial compensation to help offset any inconvenience from participation.

### Measures

#### Neuropathy

We assessed signs and symptoms of neuropathy with the Modified Total Neuropathy score (mTNS). This test evaluates sensory and motor symptoms, assessing distal muscle strength, deep tendon reflexes, light touch sensation, and vibration threshold. Scores range from 0 (indicating no neuropathy) to 24 (severe neuropathy). This modified tool is validated in pediatric children [39] and adults with cancer [40] and in cancer survivors, and has utility to both detect presence [10, 41] and monitor progression of neuropathy [42]. While a score of 1 indicates the presence of neuropathy symptoms [39], we classified neuropathy as an (mTNS score ≥ 4), consistent with the approaches used in previous studies describing associations between neuropathy signs and symptoms and functional loss in pediatric and adult ALL survivors [13, 42].

#### Sarcopenia

Sarcopenia was defined as having low lean mass, muscle weakness, or both. Lean mass was measured with dual x-ray absorptiometry (QDR4500; Hologic, Bedford, MA) in the total-body scanning mode [43–46]. The scanner was calibrated weekly with known phantoms to minimize machine drift. Relative skeletal muscle mass was calculated by dividing whole body skeletal muscle mass by height squared. Participants with relative skeletal muscle mass ≤ 1.5 standard deviations below age-, sex-, and race-specific values from the National Health and Nutrition Examination Study (NHANES) were classified as having low lean muscle mass [47]. Appendicular lean mass was calculated by summing lean mass from the arms and legs. Muscular strength assessments included isometric sitting hand grip (kg) [48] and isokinetic quadriceps strength. Isokinetic quadriceps strength (Biodex System 4, Shirley, NY) was assessed bilaterally with the participant sitting with the hips flexed at 90–100° and the back and thigh supported and secured. Knee extension strength was evaluated as maximal peak torque per kilogram of body weight from five repetitions at 60 and 180°/s; endurance was evaluated as maximal peak torque per kilogram of body weight from the last five repetitions of a ten repetition set at 300°/s. Values were converted into age- and sex-specific z-scores for analysis [49]. Participants with any hand grip, knee extension strength, or knee extension endurance z-scores ≤ 1.5 standard deviations below age-, sex-, and race-specific values were classified as having muscle weakness [50]. Muscle mass and strength ≤ 1.5 standard deviations below age-, sex-, and race-specific values are associated with mitochondrial copy number in this cohort [50].

#### Physical function

We used the Timed-Up-and-Go 3 m (TUG3m) to characterize mobility. For the TUG3m test [51], participants were instructed to stand from a 46 cm height chair, walk 3 m, turn, walk back to the chair, and sit down as fast as possible. The TUG3m test was performed twice and the fastest speed was used for analysis.

We used the 50-foot walk test to assess walking speed. For the 50-foot walk test, participants were instructed to walk 25 feet, turn around and return to the starting line as fast as possible [52]. The 50-foot walk test was performed once, and gait speed calculated as meters per second. We classified individuals with >1.3 standard deviations (lowest 10th percentile of population norms) below age and sex specific population-means on either test as having poor mobility [53].

#### Physical activity

Physical activity was self-reported using the NHANES Physical Activity Questionnaire [54]. Self-reported moderate activity was assigned an intensity of 3.5 metabolic equivalents of task (walking for pleasure) and self-reported vigorous activity an intensity of 6 metabolic equivalents of task (jogging). We assigned 1 min for each reported minute of moderate activity and 1.7 min for each minute of reported vigorous activity [55, 56]. We classified participants who met or exceeded the Centers for Disease Control and Prevention’s recommendation for physical activity (150 min per week) as physically active [55].

#### Host and treatment data

Diagnosis and treatment information was abstracted from medical records by trained abstractors and included diagnosis, age at primary malignancy diagnosis, age at assessment, chemotherapy exposures, and doses (Table 1). Sociodemographic data (age, sex and race/ethnicity), self-reported smoking status (pack years), and dietary intake according to the healthy eating index (HEI) [57] were collected via self-administered questionnaire. Body mass index (BMI) was calculated by dividing weight (measured via electronic scale) in kilograms by height (measured via stadiometer) in meters squared.

**Table 1:** Demographic, diagnosis, and treatment characteristics for study participants vs. non-participants

	Participants	Non-participants	
	*n* = 537	*n* = 282	
	*N* (%)	*N* (%)	
**Sex**			
Female	259 (48.2)	141 (50.0)	0.6303
Male	278 (51.8)	141 (50.0)	
**Race/Ethnicity**			
Non-Hispanic White	443 (82.5)	207 (73.4)	**0.0043**
Non-Hispanic Black	52 (9.7)	50 (17.7)	
Hispanic	30 (5.6)	14 (5.0)	
Asian	5 (0.9)	2 (0.7)	
Other	7 (1.3)	9 (3.2)	
**Age at primary malignancy diagnosis, Median (Min–Max)**	5.95 (0.41–18.60)	4.91 (0.29–18.73)	**0.0125**
0–4	226 (42.1)	146 (51.8)	0.0533
5–9	173 (32.2)	72 (25.5)	
10–14	99 (18.4)	43 (15.2)	
15 and above	39 (7.3)	21 (7.4)	
**Age at assessment, Median (Min–Max)**	24 (18 −54)	28 (18–52)	**0.0002**
18–29	410 (76.4)	157 (55.7)	** <0.0001**
30–39	110 (20.5)	73 (25.9)	
40–49	14 (2.6)	45 (16.0)	
50 and above	3 (0.6)	7 (2.5)	
**Body Mass Index (BMI**			
Underweight (<18.5)	17 (3.17)		
Normal (18.5–25)	208 (38.73)		
Overweight [25–30)	148 (27.56)		
Obese (≥ 30)	164 (30.54)		
**Chemotherapy agents received, within first 5 years**			
**Glucocorticoids**			
Prednisone, *n* (%)	526 (98.0)	267 (94.7)	**0.0112**
Median (Min–Max), mg/m^2^	2240.00 (326.15–19,040.00)	1120.00 (420.00–25,414.40)	
Dexamethasone, *n* (%)	297 (55.3)	190 (67.4)	**0.0004**
Median (Min–Max), mg/m^2^	10,805.40 (11.31–21,610.80)	10,805.40 (1180.67–21,770.88)	
IT hydrocortisone, *n* (%)	474 (88.3)	253 (89.7)	0.4452
Median (Min–Max)	357.45 (17.19—1115.38)	400.00 (64.86—1128.31)	
**Antimetabolites**			
IV/PO methotrexate, *n* (%)	456 (84.9)	254 (90.1)	**0.0391**
Median (Min–Max), mg/m^2^	2922.09 (40.00—83,219.57)	3097.06 (39.13—25,387.30)	
0–2922.09 mg/m^2^	226 (49.9)	108 (45.2)	0.2393
≥ 2922.09 mg/m^2^	227 (50.1)	131 (54.8)	
IT methotrexate, *n* (%)	535 (99.6)	282 (100.0)	0.5479
Median (Min–Max), mg/m^2^	180.84 (17.19—700.51)	198.48 (32.43—564.16)	
0–225 mg/m^2^	367 (69.2)	172 (64.7)	0.192
≥ 225 mg/m^2^	163 (30.8)	94 (35.3)	
6-Mercaptopurine, *n* (%)	527 (98.1)	279 (98.9)	0.5589
Median (Min–Max), mg/m^2^	36,871.27 (4956.94—94,015.22)	19,601.64 (241.18—91,451.90)	
IT cytarabine, *n* (%)	485 (90.3)	260 (92.2)	0.372
Median (Min–Max), mg/m^2^	550.82 (51.56—1673.08)	631.22 (44.12—1778.18)	
**Vincristine, *n* (%)**	537 (100.0)	281 (99.6)	0.3443
Median (Min–Max), mg/m^2^	54.92 (1.00—119.57)	56.87 (2.94—109.74)	
0–15 mg/m^2^	103 (19.4)	49 (18.4)	0.7062
15–39 mg/m^2^	54 (10.2)	32 (12.0)	
≥39 mg/m^2^	374 (70.4)	184 (69.2)	
**Anthracyclines**			
Daunorubicin, *n* (%)	452 (84.2)	246 (87.2)	0.1954
Median (Min–Max),mg/m^2^	51.41 (12.84—503.40)	50.11 (24.53—511.93)	
Doxorubicin, *n* (%)	159 (29.6)	154 (54.6)	** <0.0001**
Median (Min–Max), mg/m^2^	147.95 (23.85—291.14)	69.37 (24.44—360.00)	
**Epipodophyllotoxins**			
Etoposide, *n* (%)	266 (49.5)	81 (28.7)	** <0.0001**
Median (Min–Max), mg/m^2^	7928.00 (400.00—20,211.90)	9723.75 (268.29–19,468.18)	
Teniposide, *n* (%)	124 (23.1)	46 (16.3)	**0.031**
Median (Min–Max), mg/m2	2684.75 (150.00—7876.92)	2850.00 (597.40—8021.05)	
**Asparaginase**			
L-asparaginase, n (%)	499 (92.9)	237 (84.0)	** <0.0001**
Median (Min–Max), IU/m2	119,621.3 (5084.75–724,101.6)	121,844.0 (4000.00–641,911.9)	
Erwinia, n (%)	81 (15.1)	41 (14.5)	0.8987
Median (Min–Max), IU/m2	107,696.2 (9310.34—1,801,939)	183,368.3 (9850.75—2,468,419)	
Anthracycline dose (doxorubicin equivalents), Median (Min–Max),	52.54 (23.36—975.34)	85.64 (16.33—526.22)	
0–100 mg/m^2^	351 (75.6)	163 (68.2)	**0.039**
100–299 mg/m^2^	113 (24.3)	76 (32.8)	
Glucocorticoid dose (prednisone equivalents), Median (Min–Max),	10,830.96 (326.15—22,730.80)	11,925.40 (1120.00—25,414.40)	
0–8000 mg/m^2^	121 (23.6)	62 (24.8)	0.7126
≥ 8000 mg/m^2^	392 (76.4)	188 (75.2)	
Methotrexate dose (IV/PO/IT), Median (Min–Max),	2762.51 (18.90—83,350.00)	3178.99 (36.73—25,637.39)	
**Peripheral neuropathy by mTNS**			
No, *N*(%)	367 (68.3)		
Yes, *N*(%)	170 (31.7		
**Knee extension strength, mean (SD)**	173.40 (57.03)		
Not impaired	452 (84.2)		
Impaired	85 (15.8)		
**Knee extension endurance, mean (SD)**	77.41 (31.06)		
Not impaired	455 (84.7)		
Impaired	82 (15.3)		
**Hand grip strength, mean (SD)**	38.14 (12.62)		
Not impaired	505 (94.0)		
Impaired	32 (6.0)		
**Timed Up and Go (TUG), mean (SD**)	5.49 (1.28)		
Not Impaired	456 (84.9)		
Impaired	81 (15.1)		
**Gait speed, mean (SD)**	9.58 (1.65)		
Not impaired	370 (68.9)		
Impaired	167 (31.1)		
**Sarcopenia Status**			
Normal	319 (59.4)		
Reduced muscle strength only	102 (18.99)		
Reduced muscle mass only	88 (16.39)		
Reduced muscle strength and muscle mass	28 (5.21)		
**Physical activity**			
<150 min MVPA/week	197 (36.69)		
≥150 min of MVPA/week)	340 (63.31)		
**Smoking (Pack-years)**			
0	385 (71.7)		
0.1–19.9	143 (26.6)		
20+	2 (0.37)		

### Statistical analysis

Demographic and treatment characteristics were compared between the participants and non-participants using chi-square test, Fisher exact test, and Wilcoxon Rank-Sum Test, for whichever test was applicable. Descriptive statistics were used to characterize the study population for variables of interest, including individual health behaviors, physical performance limitations, neuropathy, and sarcopenia related outcomes*.* Modified Poisson regression was used to examine the association between treatment and peripheral neuropathy and lean muscle mass, muscle weakness, physical function limitations, and exercise behavior. The same method was applied to examine the association between peripheral neuropathy and lean muscle mass, physical function limitations and exercise behavior. Additionally, we examined the association between sarcopenia status and physical function (mobility and gait speed) and exercise behavior, using modified Poisson regression. All models were adjusted by sex, age at diagnosis, age at assessment, race and ethnicity, smoking, HEI score, and BMI. Analyses were completed in SAS version 9.4 (Cary, NC).

## Results

### Participants

**Fig. 1:**
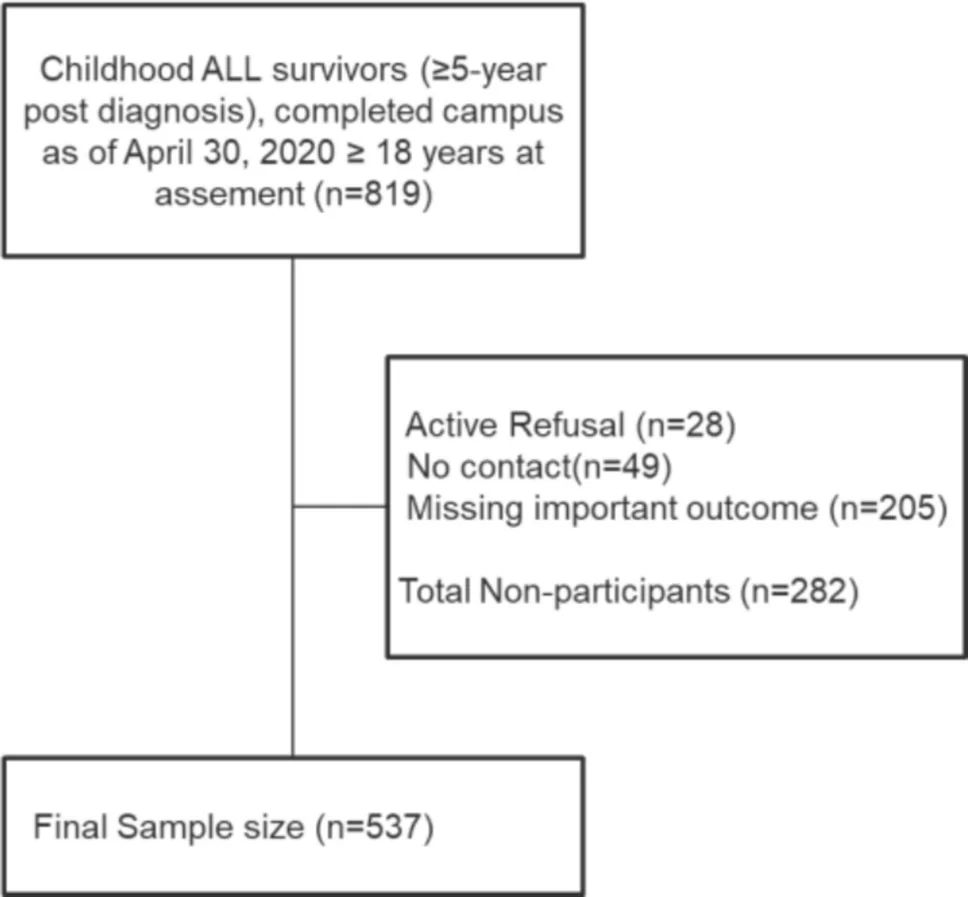
Flowchart of inclusion process

Among the 819 eligible ALL survivors, 537 (65.6%) were included in this analysis; 77 declined participation, and 205 had missing outcome data and were excluded (Fig. 1). Participants did not differ from nonparticipants by sex but were older at diagnosis, more likely to report their race/ethnicity as white, and were younger than non-participants at the time of participation. Participants had a median age of 24 years (range, 18–54 years) at assessment, and 5.95 years (range, 0.41–18.60 years) at diagnosis. Over half (58.1%) of participants had body mass index values in the overweight or obese categories. Among participants, 31.7% (*n* = 170) had peripheral neuropathy, 19.0% (*n* = 102) had reduced muscle strength, 21.6% (*n* = 116) had reduced relative lean muscle mass, and 5.2% (*n* = 28) had both reduced muscle strength and lean muscle mass (Table 1).

### Association between treatment exposures and peripheral neuropathy

Supplementary Table 1 demonstrates that vincristine dose (RR: 1.01, 95% CI 1.00 to 1.02 per 1 mg/m^2^) was associated with peripheral neuropathy in this population.

### Peripheral neuropathy and the association with sarcopenia, physical function, and physical activity

Table 2 summarizes the association between peripheral neuropathy and each of our investigated outcomes. Participants with peripheral neuropathy had a higher risk of impaired gait speed (RR: 1.31, 95% CI 1.01 to 1.70). Peripheral neuropathy was not associated with a higher risk of reduced relative lean muscle mass.

**Table 2:** Associations between peripheral neuropathy and lean muscle mass, physical performance limitations and exercise behavior

	Impaired knee extension strength		Impaired knee extension endurance		Impaired grip strength		Impaired mobility		Impaired gait speed		<150 min MVPA per week		Reduced muscle mass	
Variable	RR (95% CI)	*P*	RR (95% CI)	*P*	RR (95% CI)	*P*	RR (95% CI)	*P*	RR (95% CI)	*P*	RR (95% CI)	*P*	RR (95% CI)	*P*
**Sex**														
Male	ref (1.0)		ref (1.0)		ref (1.0)		ref (1.0)		ref (1.0)		ref (1.0)		ref (1.0)	
Female	**0.64 (0.42, 0.97)**	**0.0343**	1.05 (0.70, 1.57)	0.824	0.64 (0.32, 1.27)	0.1981	1.16 (0.78, 1.75)	0.4646	**1.32 (1.02, 1.70)**	**0.033**	**1.60 (1.27, 2.02)**	**0.0001**	0.86 (0.66, 1.14)	0.2976
**Age at diagnosis**	**1.06 (1.01, 1.11)**	**0.0174**	1.04 (0.99, 1.09)	0.0972	0.94 (0.86, 1.03)	0.1802	1.00 (0.95, 1.06)	0.9268	0.99 (0.96, 1.03)	0.6809	1.02 (1.00, 1.05)	0.1099	1.02 (0.98, 1.05)	0.3724
**Age at assessment (continuous)**	0.98 (0.95, 1.02)	0.3917	0.98 (0.94, 1.03)	0.3933	1.04 (0.97, 1.12)	0.2679	0.99 (0.95, 1.03)	0.5964	**0.96 (0.93, 0.99)**	**0.0024**	1.01 (0.99, 1.03)	0.5352	**1.04 (1.01, 1.07)**	**0.0074**
**Race**														
Non-Hispanic White	ref (1.0)		ref (1.0)		ref (1.0)		ref (1.0)		ref (1.0)		ref (1.0)		ref (1.0)	
Non-Hispanic Black	1.11 (0.64, 1.93)	0.8639	1.07 (0.62, 1.86)	0.732	1.36 (0.41, 4.52)	**0.0019**	1.58 (0.95, 2.62)	0.1093	1.30 (0.92, 1.82)	0.3312	1.11 (0.80, 1.55)	0.6898	1.27 (0.73, 2.23)	**0.026**
Other	1.17 (0.59, 2.30)		0.69 (0.25, 1.89)		**4.16 (1.89, 9.14)**		1.65 (0.83, 3.27)		1.02 (0.62, 1.68)		0.87 (0.53, 1.44)		**1.69 (1.15, 2.48)**	
**neuropathy (mTNS >/= 4)**														
No	ref (1.0)		ref (1.0)		ref (1.0)		ref (1.0)		ref (1.0)		ref (1.0)		ref (1.0)	
Yes	0.97 (0.65, 1.45)	0.8879	0.90 (0.59, 1.37)	0.6218	1.54 (0.75, 3.16)	0.2429	1.16 (0.77, 1.76)	0.4783	**1.31 (1.01, 1.70)**	**0.0406**	0.99 (0.77, 1.27)	0.9353	0.91 (0.61, 1.35)	0.6318
**Smoking (pack years)**	**1.05 (1.01, 1.10)**	**0.0133**	1.03 (0.98, 1.08)	0.194	0.95 (0.83, 1.09)	0.4967	0.98 (0.91, 1.04)	0.4652	**1.04 (1.01, 1.07)**	**0.0196**	1.00 (0.97, 1.03)	0.84	0.97 (0.93, 1.01)	0.1752
**Diet (continuous HEI score)**	1.01 (0.99, 1.02)	0.5934	1.00 (0.98, 1.02)	0.893	0.98 (0.95, 1.02)	0.3989	0.99 (0.97, 1.01)	0.2903	1.00 (0.99, 1.01)	0.5523	**0.98 (0.97, 0.99)**	**0.0001**	**0.98 (0.96, 0.99)**	**0.0018**
**BMI (continuous)**	**1.08 (1.06, 1.11)**	** <0.0001**	**1.08 (1.06, 1.11)**	** <0.0001**	0.94 (0.88, 1.01)	0.0815	**1.07 (1.04, 1.09)**	** <0.0001**	**1.03 (1.01, 1.04)**	**0.0052**	1.01 (0.99, 1.02)	0.4098	**0.73 (0.70, 0.76)**	** <0.0001**

### Sarcopenia

Table 3 summarizes the association between sarcopenia status and physical function and physical activity. Participants with impaired muscle strength had a significantly higher risk of impaired mobility (RR: 2.99, 95% CI 1.91 to 4.68) and impaired gait speed (RR: 1.45, 95% CI: 1.06 to 1.97) than those without impaired muscle strength or reduced muscle mass. Moreover, participants with both impaired muscle strength and reduced muscle mass, compared to neither, had the greatest risk of impaired mobility (RR: 3.69, 95% CI 1.75 to 7.77) and impaired gait speed (RR: 2.07, 95% CI 1.28 to 3.36). Sarcopenia status was associated with <150 min/week of moderate-to-vigorous physical activity (Table 3). Supplementary file 2 shows the number of survivors affected by one or more of the outcomes (i.e., neuropathy, muscle weakness, reduced muscle mass, and impaired mobility).

**Table 3:** Associations between sarcopenia status, physical function and exercise behavior

	Impaired mobility		Impaired gait speed		<150 min MVPA per week	
Variable	RR (95% CI)	P	RR (95% CI)	P	RR (95% CI)	P
**Sex**						
Male	ref (1.0)		ref (1.0)		ref (1.0)	
Female	1.31 (0.88, 1.93)	0.1846	**1.35 (1.04, 1.74)**	**0.022**	**1.60 (1.27, 2.01)**	**0.0001**
**Age at diagnosis**	0.99 (0.94, 1.04)	0.6489	0.99 (0.96, 1.02)	0.4386	1.01 (0.99, 1.04)	0.3187
**Age at assessment (continuous)**	0.99 (0.96, 1.03)	0.7676	**0.96 (0.94, 0.99)**	**0.0031**	1.01 (0.98, 1.03)	0.6374
**Race**						
Non-Hispanic White	ref (1.0)		ref (1.0)		ref (1.0)	
Non-Hispanic Black	1.56 (0.97, 2.53)	0.1601	1.28 (0.91, 1.79)	0.3311	1.00 (0.73, 1.38)	0.6401
Other	1.37 (0.71, 2.64)		0.94 (0.57, 1.56)		0.80 (0.50, 1.28)	
**Sarcopenia status**						
No	ref (1.0)		ref (1.0)		ref (1.0)	
reduced muscle strength only	**2.99 (1.91, 4.68)**	** <0.0001**	**1.45 (1.06, 1.97)**	**0.0052**	**1.43 (1.04, 1.95)**	** <0.0001**
reduced mass only	0.59 (0.21, 1.70)		1.27 (0.83, 1.95)		**2.25 (1.64, 3.09)**	
both	**3.69 (1.75, 7.77)**		**2.07 (1.28, 3.36)**		**2.16 (1.36, 3.42)**	
**Smoking (pack years)**	0.97 (0.91, 1.03)	0.3502	**1.04 (1.01, 1.07)**	**0.015**	1.01 (0.98, 1.04)	0.6132
**Diet (continuous HEI score)**	0.99 (0.97, 1.00)	0.1312	1.00 (0.99, 1.01)	0.5352	**0.98 (0.97, 0.99)**	**0.0012**
**BMI (continuous)**	**1.04 (1.01, 1.07)**	**0.0059**	**1.03 (1.01, 1.05)**	**0.006**	**1.03 (1.01, 1.05)**	**0.0109**

## Discussion

This study demonstrated that peripheral neuropathy is not associated with sarcopenia in childhood ALL survivors without previous cranial radiation exposure. However, both peripheral neuropathy and sarcopenia are associated with impaired physical function (mobility and gait speed) and exercise behavior.

Although previous data indicate that childhood ALL survivors exhibit a reduced sural sensory nerve action potential amplitude along with prolonged latency of compound muscle action potentials, suggesting suboptimal peripheral nerve function and diminished number of active axons and motor units, we did not find an association between neuropathy and features of sarcopenia. Theoretically, abnormal movement feedback and the loss of motor units should contribute to skeletal muscle atrophy and muscle weakness [10, 13]. This theory was not supported by our data. We did observe a significant association between neuropathy and impaired gait speed, with affected participants demonstrating a 31% increased risk of gait impairment. This aligns with previous studies indicating that peripheral neuropathy contributes to functional limitations and decreased mobility in childhood cancer survivors [58]. Our data suggests that while peripheral neuropathy may directly affect neuromuscular control and mobility, its impact on muscle mass and strength may be less pronounced or mediated by other factors such as health behaviors (i.e., diet, physical activity, and smoking).

Almost one fifth of the survivors in this cohort exhibited markers of sarcopenia (i.e., low lean muscle mass, muscle weakness, or both). Furthermore, sarcopenia emerged as a strong predictor of poor physical function (mobility and gait speed). Participants with impaired muscle strength exhibited nearly three times the risk of impaired mobility and a 45% increased risk of impaired gait speed compared with those without strength impairments, and those with both impaired muscle strength and reduced muscle mass demonstrated even greater risks of mobility impairment and gait dysfunction. In the general population, sarcopenia becomes a significant issue around the age of 50, with an accelerated decline occurring after 60–70 years. Comparatively, this cohort of survivors was a mean of 28 years old at the time of assessment—suggesting premature aging [59]. Sarcopenia has been linked to increased risk for fall and fractures [22, 60], impaired mobility [61], and difficulty with activities of daily living [23, 62], and loss of independence [24]. Sarcopenia is further associated with cardiac disease [25], respiratory disease [63], and cognitive impairment [26], contributing to reduced quality of life [27] and premature death [28].

Given these health concerns and our results, early intervention strategies are critical for mitigating the impact of neuropathy and sarcopenia on overall health in survivors of childhood ALL [64, 65]. Several small studies indicate that peripheral neuropathy and sarcopenia are responsive to intervention in childhood cancer survivors [66–68]. Tanner et al. [69] reported that physical therapy during treatment improved bilateral coordination, running speed, and muscle strength compared with no physical therapy, approximately 20 months after treatment ended in 30 survivors of childhood ALL. Similarly, Krull et al. [70] showed that resistance training—regardless of protein supplementation—can improve muscle mass and strength in adult survivors of childhood cancer with low muscle mass [70]. These results suggest that exercise interventions can indeed counteract sarcopenia in childhood cancer survivors. Additional studies are needed to verify these promising results in survivors of childhood ALL.

### Limitations

This study has several limitations. First, the cross-sectional design precludes causal inferences regarding the relationships between neuropathy, sarcopenia, health behaviors, and functional impairments. Longitudinal studies are needed to assess the progression of these conditions over time. Second, our reliance on self-reported physical activity levels may introduce reporting bias. Future research should incorporate objective measures of physical activity, such as accelerometry, to strengthen the validity of these findings. Finally, our sample predominantly comprised white participants, which may limit the generalizability of results to more diverse populations.

### Clinical implications

These findings have important clinical implications for the long-term care of survivors of childhood ALL. Although peripheral neuropathy was not associated with sarcopenia, both conditions were independently linked to impaired mobility and gait speed, supporting the need for routine functional assessment in survivorship care. Evaluation of muscle strength, lean mass, and gait performance may facilitate early identification of survivors at risk for functional decline and premature aging. The observed prevalence of sarcopenia-related features in this young cohort highlights a window for early intervention. Given evidence that exercise-based strategies can improve neuromuscular function and muscle health in childhood cancer survivors, timely referral to physical therapy and resistance training programs may mitigate functional limitations, reduce future morbidity, and support long-term independence.

## Conclusion

Neuropathy and sarcopenia significantly impact physical function in childhood ALL survivors, with impaired muscle strength emerging as a key determinant of mobility limitations. However, no association was observed between peripheral neuropathy and aspects of sarcopenia. These findings emphasize the importance of targeted interventions, including exercise, to improve functional outcomes in this vulnerable population and provide insights into the long-term toxicity of therapy which can inform future therapeutic ALL trial deintensification efforts. Future research should focus on developing evidence-based rehabilitation strategies to enhance long-term survivorship care.

## Supplementary Information

Below is the link to the electronic supplementary material.

ESM1 (XLSX 13.6 KB)

ESM 2 (PNG 687 KB)

High Resolution Image (TIF 102 KB)

## Data Availability

Data used for these analyses are publicly available at Visualization Community | St. Jude Cloud. Specific data for these analyses are posted on Zenodo.com.
